# Potency of cashew nut shell liquid in rumen modulation under different dietary conditions and indication of its surfactant action against rumen bacteria

**DOI:** 10.1186/s40781-017-0150-8

**Published:** 2017-11-24

**Authors:** Seongjin Oh, Yasuyuki Suzuki, Shusuke Hayashi, Yutaka Suzuki, Satoshi Koike, Yasuo Kobayashi

**Affiliations:** 1 Graduate School of Agriculture, Hokkaido University, Sapporo, Hokkaido 060-8589 Japan; 2 Laboratory of Animal Function and Nutrition, Graduate School of Agriculture, Hokkaido University, Sapporo, 060-8589 Japan

**Keywords:** Cashew nut shell liquid, Forage to concentrate ratio, Rumen bacteria, Rumen modulation

## Abstract

**Background:**

Cashew nut shell liquid (CNSL) is an agricultural byproduct containing alkylphenols that has been shown to favorably change the rumen fermentation pattern only under experimentally fixed feeding conditions. Investigation of CNSL potency in rumen modulation under a variety of feeding regimens, and evidence leading to the understanding of CNSL action are obviously necessary for further CNSL applications. The objective of this study was to evaluate the potency of CNSL for rumen modulation under different dietary conditions, and to visually demonstrate its surfactant action against selected rumen bacteria.

**Methods:**

Batch culture studies were carried out using various diets with 5 different forage to concentrate (F:C) ratios (9:1, 7:3, 5:5. 3:7 and 1:9). Strained rumen fluid was diluted with a buffer and incubated with each diet. Gas and short chain fatty acid (SCFA) profiles were characterized after 18 h incubation at 39 °C. Monensin was also evaluated as a reference additive under the same conditions. Four species of rumen bacteria were grown in pure culture and exposed to CNSL to determine their morphological sensitivity to the surfactant action of CNSL.

**Results:**

CNSL supplementation decreased total gas production in diets with 5:5 and 3:7 F:C ratios, whereas the F:C ratio alone did not affect any gas production. Methane decrease by CNSL addition was more apparent in diets with 5:5, 3:7, and 1:9 F:C ratios. An interactive effect of CNSL and the F:C ratio was also observed for methane production. CNSL supplementation enhanced propionate production, while total SCFA production was not affected. Monensin decreased methane production but only in a diet with a 1:9 F:C ratio with increased propionate. Studies of pure cultures indicated that CNSL damaged the cell surface of hydrogen- and formate-producing bacteria, but did not change that of propionate-producing bacteria.

**Conclusion:**

CNSL can selectively inhibit rumen bacteria through its surfactant action to lead fermentation toward less methane and more propionate production. As CNSL is effective over a wider range of dietary conditions for such modulation of rumen fermentation in comparison with monensin, this new additive candidate might be applied to ruminant animals for various production purposes and at various stages.

## Background

Enteric methane is mainly produced in the gastrointestinal tract of ruminant animals during their feed utilization, and is closely related to loss of energy in feed stuff [1]. Many studies have been carried out using feed additives to modulate rumen fermentation in order to improve feed efficiency, and the quantity and quality of animal products such as meat and milk [2–4]. Although ionophore antibiotic additives, represented by monensin, have been used in many countries, the use of these additives has been gradually prohibited due to concerns regarding public health [5, 6]. Natural additive sources (e. g. plant extracts, essential oils etc.) appear to be alternative additives for rumen modulation [7–9].

Cashew nut shell liquid (CNSL) is a byproduct of the cashew nut industry and has been used as a material for products including paints, lacquers, coatings, and other products [10]. CNSL was recently evaluated as a potential additive source for ruminants, because it contains alkylphenols (anacardic acid, cardanol and cardol) that can favorably modify rumen microbiota and fermentation [11]. Of these phenolics, anacardic acid is thought to have the highest antimicrobial activity [12, 13], and this notion was also supported by recent studies using anacardic acid and its source materials CNSL and ginkgo fruit [11, 14]. Favorable shifts of microbiota and fermentation were, however, confirmed in in vitro evaluations only under fixed single dietary conditions. To employ this potent additive candidate in wider applications, CNSL needs to be tested under various dietary conditions based on the purposes and stages of animal production.

The mode of inhibition of bacteria by CNSL is considered to be the surfactant action of anacardic acid that physically breaks down the cell surface of bacteria. Therefore, Gram-negative bacteria that possess an outer membrane often exhibit tolerance to such a surfactant [15, 16]. Although the inhibitory concentration of CNSL, ginkgo fruit and their component phenolics against rumen bacteria were determined in previous studies [11, 14], no direct observation of the cell morphology of rumen bacteria that had been exposed to CNSL has been made.

Evaluation of the above issues (the potency of CNSL depending on dietary conditions and the mode of action of CNSL) is important for proposing CNSL application strategies in various feeding regimens for ruminants based on an understanding of key CNSL mechanisms. The objectives of the present study were, therefore, to evaluate the potency of CNSL under various dietary conditions, and to determine its surfactant action against selected rumen bacteria that are responsible for rumen modulation. To evaluate the effectivity of CNSL for modulating rumen fermentation, monensin was also employed in in vitro evaluation in the same manner.

## Methods

### Rumen fluid used

Rumen content was taken from two ruminally cannulated Holstein dry cows at the experimental farm of Hokkaido University, Sapporo, Japan. The donor cows were fed twice daily (08:00 and 17:00) with a 50% concentrate (Monster 18; Mercian, Tokyo, Japan) and 50% orchardgrass hay diet, which contained 18.2% crude protein, 41.2% neutral detergent fiber and 2.16 Mcal metabolizable energy/kg on a dry matter basis. The obtained rumen contents from cows were equally mixed, placed into a bottle flushed with N_2_ gas, which was then transferred to the laboratory within 30 min. The rumen content was then strained through 2 layers of surgical gauze for experimental use.

### Batch culture

Table 1 shows the chemical composition of the diet and the CNSL used. The experimental substrate was a mixture of 0.2 g orchardgrass hay and concentrate, with five forage to concentrate (F:C) ratios (9:1, 7:3, 5:5. 7:3 and 1:9). These materials were ground by using a 1 mm sieve-attached cutter mill. CNSL was obtained from Idemitsu Kosan Co. Ltd. (Sodegaura, Chiba, Japan). Monensin (monensin sodium salt) was purchased from Sigma-Aldrich (Saint Louis, MO, USA). Both additives were evaluated for their potency for modulation of rumen fermentation by batch culture experiments using rumen fluid diluted (2×) with McDougall’s buffer [17] and the above substrates. Supplementation levels of CNSL and monensin were 500 and 5 μg/mL, respectively. It has been experimentally defined that these levels do not negatively influence total short chain fatty acid (SCFA) production [11, 18] and these levels were therefore recommended in the present study. Each additive was dissolved in 99.5% ethanol, added to empty culture tubes and left overnight to evaporate the ethanol. As a control, ethanol was added and treated in the same manner. Diluted rumen fluid was added to each tube containing substrate with or without each additive, and the head space of the tubes was flushed with N_2_ gas, then sealed with a butyl rubber stopper and a plastic screw cap, and incubated at 39 °C for 18 h, as done by Watanabe et al. [11]. Four replicates were incubated for each treatment. After incubation, the total gas of the head space was measured using a needle-attached pressure gauge (Aϕ60B; GL Science, Tokyo, Japan) and was also employed for gas composition analysis. The culture liquid was sampled and kept at −30 °C for SCFA analysis.

**Table 1:** Chemical composition of the experimental diet and the cashew nut shell liquid

Content	Hay	Concentrate	CNSL
	g/kg		
Dry matter	971	969	983
	g/kg DM		
Crude protein	74	203	15
Crude ash	63	61	12
Ether extract	9	97	942
Neutral detergent fiber	743	345	–
Acid detergent fiber	448	134	–
Water soluble carbohydrate	112	294	–
Nitrogen free extract	–	–	31
Ether extract fractions, %			
Total alkylphenols	–	–	92.7
Total anacardic acid	–	–	62.3
monoenoic (15:1)	–	–	28.9
dienoic (15:2)	–	–	8.8
trienoic (15:3)	–	–	24.6
Total cardanol	–	–	8.9
monoenoic (15:1)	–	–	2.9
dienoic (15:2)	–	–	1.5
trienoic (15:3)	–	–	4.5
Total cardol	–	–	21.5
monoenoic (15:1)	–	–	3.1
dienoic (15:2)	–	–	4.3
trienoic (15:3)	–	–	14.1
Others^a^	–	–	7.3

^a^unidentified fractions

### Pure cultures

The bacteria used were *Ruminococcus flavefaciens* C94, *Butyrivibrio fibrisolvens* D1, *Megasphaera elsdenii* LC1 and *Selenomonas ruminantium*.GA192, all of which are a type strain of each species. These bacteria were anaerobically cultivated in rumen fluid containing medium [14]. When each bacterium was grown to the exponential phase, ethanol (control) or CNSL was added (200 μg/mL final concentration) and incubation was continued for 5 h. The culture was then analyzed using scanning electron microscopy (SEM) as follows. Bacterial samples were washed with 20 mM K phosphate buffer (pH 7.2), soaked in 2.5% glutaraldehyde in K phosphate buffer and then fixed with 1% osmic acid in K phosphate buffer. The samples were then dehydrated using different ethanol concentrations of 50, 70, 90, and 99.5%. The next step was dehydration with iso-amylacetate and a critical point drier (HCP-2, Hitachi, Japan). The sample was then coated with gold-paradium by ion spatter and was observed using a high resolution scanning electron microscope (JSM-6301F, Japan Electron, Tokyo, Japan).

### Chemical analysis

Gas and SCFA analyses were carried out essentially as described by Oh et al. [14]. Head space gas was analyzed using gas chromatography (GC-8A; Shimadzu, Kyoto, Japan) with attached parallel columns, Porapak Q (Waters, Milford, MA) and Molecular Sieve 13X (Restek, Bellefonte, PA), and a thermal conductivity detector. Flame ionization detector-attached gas chromatography (GC-14B; Shimadzu, Kyoto, Japan) was used for SCFA analysis, using a fused silica capillary column (ULBON HR-20 M, 0.53 mm i.d. × 30 m length, 3.0 μm film; Shinwa, Kyoto, Japan).

### Statistical analysis

The data (*n* = 4) of rumen parameters obtained from two batch culture studies (experiment 1: control vs. CNSL, and experiment 2: control vs. monensin) were individually subjected to analysis of variance using the general linear model procedure of SPSS (version 16.0 J, Tokyo, Japan). Tukey’s method was employed for multiple comparison across 5 different F:C ratios and 2 treatments (control vs. CNSL or monensin). Statistical significance was declared at *P* < 0.05.

## Results

### Gas profiles

The effect of CNSL or monensin supplementation on rumen gas production from diets with different F:C ratios is shown in Table 2. CNSL supplementation decreased total gas production from diets with 5:5 and 3:7 F:C ratios (*P* < 0.05), while the F:C ratio alone did not affect any gas production. CNSL strongly inhibited methane production, especially from diets with 5:5 (46%), 3:7 (46%), and 1:9 (51%) F:C ratios (*P* < 0.05). An interactive effect between CNSL and diet (F:C ratio) was also observed for methane production (*P* < 0.01). No specific accumulation of hydrogen was observed. Monensin supplementation did not alter total gas, CO_2_ or hydrogen production. Methane was significantly decreased by monensin only for a diet with a 1:9 F:C ratio, showing 46% reduction (*P* < 0.05).

**Table 2:** Effect of cashew nut shell liquid and monensin supplementation under different dietary conditions on in vitro gas production

Gases mL/culture	Forage to concentrate ratio										*P*-value		
	9:1		7:3		5:5		3:7		1:9				
	Control	Treatment	Control	Treatment	Control	Treatment	Control	Treatment	Control	Treatment	Additive	Diet	A × D
Cashew nut shell liquid													
Total gas	4.00^abc^	3.58^bc^	4.84^ab^	4.27^abc^	4.87^ab^	3.32^c^	5.08^a^	3.56^bc^	4.81^ab^	3.69^abc^	<0.001	0.173	0.213
CO_2_	3.44^ab^	2.96^ab^	3.95^ab^	3.72^ab^	3.93^ab^	2.81^b^	4.08^a^	3.01^ab^	3.78^ab^	3.18^ab^	<0.001	0.200	0.349
CH_4_	0.54^bc^ (100)	0.60^bc^ (111)	0.88^abc^ (100)	0.54^c^ (61)	0.93^ab^ (100)	0.50^c^ (54)	0.98^a^ (100)	0.53^c^ (54)	1.02^a^ (100)	0.50^c^ (49)	<0.001	0.103	0.008
H_2_	0.01	0.02	0.01	0.02	0.01	0.01	0.01	0.01	0.01	0.01	0.744	0.466	0.428
Monensin													
Total gas	4.62	4.44	5.75	4.80	5.47	4.82	5.17	4.68	5.31	4.39	0.009	0.442	0.742
CO_2_	3.61	3.30	4.47	3.71	4.03	3.69	3.79	3.66	3.96	3.50	0.059	0.535	0.875
CH_4_	1.13^ab^ (100)	1.14^ab^ (101)	1.27^a^ (100)	1.08^ab^ (85)	1.43^a^ (100)	1.12^ab^ (85)	1.36^a^ (100)	1.01^ab^ (78)	1.35^a^ (100)	0.73^b^ (54)	<0.001	0.295	0.092
H_2_	0.01	0.01	0.02	0.01	0.01	0.01	0.01	0.01	0.01	0.01	0.001	0.642	0.949

Values in parenthesis are relative percentages of methane production in treatment to that in control

A × D indicates interaction between additive and diet effects

^a-c^Means within a row with different superscripts significantly differ (*P* < 0.05)

### SCFA profiles

The effect of CNSL or monensin supplementation on in vitro rumen SCFA profiles is shown in Table 3. The total concentration of SCFA was generally not affected by CNSL, although an increase in SCFA by CNSL was observed for a diet with a 5:5 F:C ratio (*P* < 0.05). Proportions of acetate and butyrate were decreased by CNSL supplementation (*P* < 0.001), while that of propionate was increased under all dietary conditions tested (*P* < 0.001). The enhancement of propionate was more pronounced for diets with 5:5, 3:7 and 1:9 F:C ratios. In terms of the molar proportions of SCFA, additive effect, diet effect and their interactive effect were significant (*P* < 0.001). Regarding monensin addition, the total SCFA level was not affected by monensin in comparison with control, whereas the proportion of acetate was decreased in diets of all F:C ratios (*P* < 0.05), except for the 9:1 ratio. An increase in the proportion of propionate and a decrease in the proportion of butyrate were found with high concentrate diets (3:7 and 1:9 F:C ratios) (*P* < 0.05). A diet effect was therefore observed for total SCFA, propionate and butyrate levels and proportions of acetate and propionate (*P* < 0.001).

**Table 3:** Effect of cashew nut shell liquid and monensin supplementation under different dietary conditions on in vitro short chain fatty acid production

Parameters	Forage to concentrate ratio										*P*-value		
	9:1		7:3		5:5		3:7		1:9				
	Control	Treatment	Control	Treatment	Control	Treatment	Control	Treatment	Control	Treatment	Additive	Diet	A × D
	mmol/dL												
Cashew nut shell liquid													
Total SCFA	9.57^c^	9.81^bc^	9.64^bc^	10.67^ab^	10.26^bc^	11.36^a^	10.71^ab^	10.71^ab^	10.67^ab^	10.49^abc^	0.004	<0.001	0.019
Acetate	6.44^ab^	6.41^ab^	6.40^ab^	6.70^ab^	6.72^a^	6.83^a^	6.82^a^	6.35^bc^	6.74^a^	6.12^b^	0.078	0.048	0.003
Propionate	1.64^e^	2.11^cde^	1.71^de^	2.60^b^	1.90^cde^	3.32^a^	2.13^bcd^	3.30^a^	2.21^bc^	3.46^a^	<0.001	<0.001	<0.001
n-Butyrate	1.13^c^	0.99^de^	1.20^bc^	1.07^cd^	1.30^ab^	0.94^de^	1.40^a^	0.84^ef^	1.36^a^	0.73^f^	<0.001	0.011	<0.001
Monensin													
Total SCFA	8.08^b^	7.96^b^	8.26^b^	8.29^b^	8.71^ab^	9.03^ab^	8.69^ab^	8.80^ab^	9.41^a^	8.73^ab^	0.650	<0.001	0.280
Acetate	5.20^ab^	5.00^ab^	5.15^ab^	4.92^ab^	5.24^ab^	5.16^ab^	5.07^ab^	4.81^ab^	5.38^a^	4.69^b^	0.002	0.416	0.228
Propionate	1.82^cd^	1.78^d^	1.92^cd^	2.16^bcd^	2.19^bcd^	2.65^ab^	2.33^bc^	2.91^a^	2.64^ab^	2.95^a^	<0.001	<0.001	0.093
n-Butyrate	0.82^d^	0.90^cd^	0.95^bcd^	0.99^abcd^	1.03^abc^	1.04^abc^	1.09^ab^	0.92^bcd^	1.16^a^	0.93^bcd^	0.029	<0.001	0.001
	molar %												
Cashew nut shell liquid													
Acetate	67.28^a^	65.35^b^	66.39^ab^	62.73^c^	65.48^b^	60.13^d^	63.72^c^	59.30^de^	63.22^c^	58.37^e^	<0.001	<0.001	<0.001
Propionate	17.17^f^	21.46^d^	17.74^f^	24.34^c^	18.47^ef^	29.16^b^	19.90^de^	30.67^b^	20.72^d^	32.99^a^	<0.001	<0.001	<0.001
n-Butyrate	11.80^b^	10.03^c^	12.43^ab^	10.05^c^	12.63^ab^	8.31^d^	13.08^a^	7.87^d^	12.72^ab^	6.90^e^	<0.001	<0.001	<0.001
Monensin													
Acetate	64.21^a^	62.53^a^	62.33^ab^	59.33^cd^	60.12^bc^	57.19^d^	58.34^cd^	54.58^e^	57.23^d^	53.78^e^	<0.001	<0.001	0.280
Propionate	22.51^de^	22.27^e^	23.21^de^	26.00^cde^	25.02^cde^	29.29^bc^	26.67^cd^	33.05^ab^	28.11^c^	33.79^a^	<0.001	<0.001	0.007
n-Butyrate	10.19^c^	11.27^abc^	11.52^abc^	11.87^abc^	11.87^abc^	11.49^abc^	12.51^a^	10.47^bc^	12.37^ab^	10.57^bc^	0.034	0.119	0.001

A × D indicates interaction between additive and diet effects

^a-f^Means within a row with different superscripts significantly differ (*P* < 0.05)

### Bacterial morphology

**Fig. 1:**
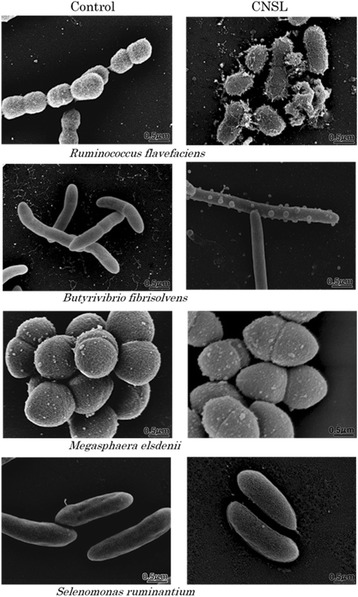
Morphological changes of rumen bacteria induced by cashew nut shell liquid. Each bacterium was exposed to ethanol (control) or cashew nut shell liquid (CNSL) for 5 h at log exponential growth phase

Figure 1 shows the morphology of 4 selected rumen bacterial species with or without exposure to CNSL, as determined using SEM. The cell surface of *Ruminococcus flavefaciens*, a hydrogen-producing fibrolytic bacterium, suffered heavy damage following CNSL exposure. The damaged cells of this bacterium had a hairy surface in appearance and some cells were completely broken and burst. Exposure of the bacterium *Butyrivibrio fibrisolvens*, which is a hydrogen and butyrate producer, to CNSL resulted in the formation of bubble-like bumps on the cell surface. The length of this bacterium also increased and cell division seemed to be inhibited. Thus, the surface structure of both of these bacterial species was apparently changed by exposure to CNSL. By contrast, there were no morphological changes in the cell surface or in cell size following exposure of the two propionate-producing bacterial species, *Megasphaera elsdenii* and *Selenomonas ruminantium* to CNSL.

## Discussion

In the present study, responses of rumen fermentation to CNSL under a variety of dietary treatments were investigated. As a reference additive, monensin was tested in the same manner. The present batch culture studies showed that both additives similarly changed gas and SCFA production, which involved a decrease in methane, acetate and butyrate production, and an increase in propionate production. However, the extent of these changes was greater for CNSL than for monensin addition, at the recommended level for each additive, and the effective range of diet that was effected was broader in CNSL than in monensin addition (Tables 2 & 3). The present preliminary evaluation of the effect of CNSL addition on the modulation of rumen fermentation supports the notion that not only can CNSL be used in a fixed condition as indicated in a previous study [11] but that it may also be used under a wide range of dietary conditions. This finding is advantageous when taking into consideration the potential use of CNSL addition in the practice of ruminant feeding.

Although the reason why there is a broad CNSL efficacy among diets is not apparent, the mode of action of CNSL is simple (via surfactant action, see below) but strong in comparison with other additives including monensin. This activity could lead to clear microbial selection followed by favorable fermentation changes in terms of methane mitigation and propionate enhancement. In addition, CNSL addition may prevent rumen metabolic disorders such as lactic acidosis and feedlot bloat when a high grain diet is fed, because CNSL inhibits the growth of the lactate-producing *Streptococcus bovis* and decreases the viscosity of rumen fluid [11]. Applying these characteristics to practical use, CNSL might be a useful additive for beef cattle receiving a high grain diet. However, whether the efficacy of CNSL is long-lasting when continuously fed to animals for a long period of time [19], remains to be assessed.

Modes of action of CNSL and monensin in rumen modulation have been discussed in previous studies, in which CNSL was shown to have a surfactant activity [11–14] and monensin was shown to have an ionophore activity [18, 20]. However, no direct evidence of the surface acting action of CNSL has been shown for bacteria, especially for rumen bacteria. We observed the morphology of representative rumen bacterial species exposed to CNSL using SEM. This analysis clearly proved that the cell surface of two species of rumen bacteria that produce hydrogen and formate was physically interfered with upon CNSL exposure, while the cell surface of propionate-producing species did not show any change at all upon CNSL exposure (Fig. 1). These observations can be explained by the fact that the latter 2 species (*S. ruminantium* and *M. elsedenii*) possess an outer membrane that protects the cells from surfactant action, while the former 2 species (*R. flavefaciens* and *B. fibrisolvens*) lack such a membrane [21, 22], which means that the surfactant CNSL can directly act against the bacterial cell surface to physically break it. According to Watanabe et al. [11], the minimum inhibitory concentrations of CNSL (μg/ml) for the growth of these bacteria are 1.56 (*R. flavefaciens*), 3.13 (*B. fibrisolvens*), and >50 (*M. elsedenii* and *S. ruminantium*). These values apparently correspond to the physical sensitivity/strength of each bacterium against the surfactant action of CNSL as shown by the present microscopic observations. This study provides the first and direct visual evidence for the surfactant action of CNSL.

Theoretically, it is considered that other bacterial species are influenced by CNSL in the same manner, based on the presence and/or absence of an outer membrane. This simple mode of action of CNSL could successfully alter rumen microbiota, thereby shifting metabolic hydrogen flow and leading to greater propionate production via fumarate and acrylate pathways [23, 24]. *Ruminococcus flavefaciens* and *Butyrivibrio fibrisolvens* are known to be fiber degraders and their end-products such as H_2_, formate and acetate, consistently contribute to methane production in the rumen [25, 26]. Additionally, other rumen hydrogen and/or formate producers including *Treponema bryantii* were considered to be sensitive to CNSL and their abundance actually decreased both when CNSL was supplemented to the continuous culture RUSITEC [11] and also when CNSL was fed to cattle [19]. These data also explain why rumen methane production is depressed by CNSL.

As far as CNSL does not completely inhibit specific group of bacteria in the rumen, the extent of fermentation change is essentially affected by type of substrate. Therefore, the reason for more pronounced effect of CNSL under high grain diets could be explained as follows. Bacteria involved in propionate production can be selected more apparently by CNSL under the presence of the substrate concentrate, while hydrogen- or formate-producing bacteria, sensitive to CNSL, can be more suppressed with scanty of the substrate forage. As a result, shift of rumen microbiota became more demonstrable, leading to more apparent fermentation changes by CNSL. However, further evaluations in animal feeding experiments are necessary for confirming the potency of CNSL as a rumen modulating agent.

## Conclusion

CNSL altered the in vitro rumen fermentation pattern towards less methane and more propionate production under 5 different dietary conditions tested. CNSL supplementation showed a greater extent of rumen modulation and showed efficacy over a wider coverage of diet composition in comparison with supplementation with the ionophore monensin. The surfactant action of CNSL against specific rumen bacteria was visually indicated to be the main cause of rumen modulation. Feeding studies are required to practically assess the potency of CNSL in ruminants on various diets.

## Abbreviations

CNSL: Cashew nut shell liquid
F:C ratio: Forage to concentrate ratio
SCFA: Short chain fatty acid
SEM: Scanning electron microscopy
